# Clinical study on traditional Chinese medicine fumigation for improving shoulder joint function after ultrasound-guided hypertonic dextrose injection in patients with rotator cuff injury

**DOI:** 10.3389/fmed.2026.1896206

**Published:** 2026-08-19

**Authors:** Yunwu Fan, Wenping Cao, Jianfeng Pu, Yeting Chen, Ye Cao

**Affiliations:** 1 Department of Pain Medicine, Zhangjiagang Second People's Hospital, Zhangjiagang, Jiangsu, China; 2 Department of Acupuncture, Zhangjiagang Second People's Hospital, Zhangjiagang, Jiangsu, China

**Keywords:** rotator cuff injury, ultrasound guidance, hypertonic dextrose injection, traditional Chinese medicine fumigation, randomized controlled trial

## Abstract

**Aim:**

This study was aimed at exploring the efficacy and safety of traditional Chinese medicine (TCM) fumigation in improving shoulder joint function after ultrasound-guided hypertonic dextrose injection in patients with rotator cuff injury.

**Methods:**

One hundred and eighty-four subjects with rotator cuff injury were randomized into two equal groups: an experimental group and a control group. The control group only received the ultrasound-guided hypertonic dextrose injection, while the experimental group received TCM fumigation treatment following the injection. The two groups were compared for their Constant-Murley Shoulder (CMS) score, Modified University of California at Los Angeles Shoulder Rating Scale (Modified UCLA) score, Visual Analog Scale (VAS) scores and safety before enrollment as well as 2 and 6 months after the completion of the treatment course. The clinical efficacy of the treatment was evaluated 6 months after the end of the course.

**Results:**

The pretreatment scores of the two groups did not show any statistically significant differences. However, at the end of the 2 months of the treatment, the experimental group showed higher CMS scores [Mean Difference (MD) = 6.83, 95% CI (4.18, 9.48), Cohen's *d* = 0.75, *t* = 5.09, *P* < 0.001]; lower VAS scores [Median Difference (MD) = −0.70, 95% CI (−1.00, −0.30), *r* = 0.29, *Z* = −3.92, *P* < 0.001]; and higher modified UCLA scores [MD = 2.07, 95% CI (0.75, 3.40), Cohen's *d* = 0.46, *t* = 3.09, *P* = 0.002], than the control group. At the end of 6 months of the treatment, no statistically significant differences were noted in the scores in the two groups. There were no statistically significant differences in the rates of marked effect and total rate of effectiveness [Rate Difference (RD) = 0.04, 95% CI (−0.09, 0.18), ϕ = 0.05, χ^2^ = 0.39, *P* = 0.533], and [RD = 0.01, 95% CI (−0.10, 0.12), ϕ = 0.01, χ^2^ = 0.04, *P* = 0.848]. The two groups did not show any significant difference with respect to safety in terms of the frequency of adverse events.

**Conclusion:**

Compared with ultrasound-guided hypertonic dextrose injection alone, a combined treatment plan where the injection is followed by TCM fumigation can significantly improve the shoulder joint function and reduce the pain score of subjects in the short term, with good safety. However, there were no significant differences in the long-term efficacy of the two methods.

**Clinical Trials Registration:**

(http://www.chictr.org.cn), identifier [ChiCTR2100050251].

## 1 Introduction

Rotator cuff injury results from the tearing of the tendons forming the rotator cuff due to acute injury or repeated chronic strain, causing pain in the shoulder joint and restriction of active elevation and abduction, without any obvious limitation in the range of passive movement. In cases of chronic injury, patients may develop varying degrees of atrophy of the periscapular muscles and secondary contracture of the shoulder joint. Among the tendons of the rotator cuff, the supraspinatus tendon is the most vulnerable, accounting for 70.8% of all injuries, followed by the infraspinatus tendon and the subscapularis tendon; injuries to the teres minor tendon are rare (1).

Studies have shown that the occurrence of rotator cuff injury increases with age, with the incidence being 9.7% in persons under 20 years old and 62% in those over 80 years old (2). Similarly, a study from the UK found that 22.2% of women aged between 64 and 87 years sustained rotator cuff injuries (3).

Rotator cuff injuries are treated with both surgical and conservative approaches; however, surgical treatment has not been shown to be superior to conservative treatment, and over time, both treatment methods improve symptoms to some extent (4, 5). Surgery is traumatic, costly, and carries potential risk of infection and/or the retearing of the rotator cuff (4). Therefore, patients are less willing to undergo surgical treatment. A recent study found that only 25% of patients ultimately require surgical treatment, while the rest can achieve relatively satisfactory outcomes through conservative treatment (1).

Conservative treatment for rotator cuff injuries includes injection therapy; non-steroidal anti-inflammatory drugs; and exercise and physical therapy (4, 6); as well as traditional Chinese medicine (TCM) treatment methods such as acupuncture, massage, acupoint application, moxibustion, oral drugs, and fumigation (7, 8). As one of the injection therapies, prolotherapy has favorable curative effects on various tendon and joint conditions. Hypertonic dextrose injection is the most commonly used (9), and ultrasound guidance can further improve the accuracy of reaching the target and treatment outcome (10).

In 2014, Zhang et al (11) pointed out that TCM fumigation promotes blood circulation to eliminate blood stasis and dredging tendons and collaterals, thereby relieving pain and joint function in patients with rotator cuff injuries. For improved treatment outcomes in patients with rotator cuff injuries, since October 2021, we have employed a combined approach of administering ultrasound-guided hypertonic dextrose injection followed by TCM fumigation to treat rotator cuff injuries. In this study, we explore the safety and efficacy of this combined treatment strategy.

## 2 Materials and methods

### 2.1 Research design

This study was designed as a single-center, prospective, randomized, parallel-controlled, open-label clinical trial comprising an experimental group and a control group. The study was conducted in accordance with the ethical principles and restrictions of the Declaration of Helsinki (12). The research protocol was approved by the Medical Ethics Committee of Zhangjiagang Second People's Hospital (Approval No.: ZEY-2021015) and was registered at the Chinese Clinical Trial Registry in August 2021 (Registration No.: ChiCTR2100050251).

### 2.2 Sample size calculation

The primary outcome indicator used in this study was the Constant-Murley Shoulder (CMS) Score (13). We conducted a preliminary pre-test, wherein the difference in the mean CMS scores between the experimental group and the control group was estimated to be approximately 7.0 points at about 2 months after the end of the treatment course, with the standard deviations of the two groups being both 9.0 points. Considering the equal-ratio design of the experimental group and the control group, the PASS 15.0 software was used to estimate the sample size for the superiority test of two means. The significance level of α was 0.05 (two-sided test), power 1-β was 0.8, and the superiority margin Δ was 3.5. The estimated effective sample size required for each group was *n* = 83. To account for possible dropout of subjects, as per our previous experience, the sample size of each group was increased by 10% on the basis of the original estimate, adjusted to *n* = 92. The required sample size was thus determined to be 184 subjects.

### 2.3 Subject setting

The subjects recruited in our study were patients with shoulder and upper limb pain who visited the Acupuncture Department or Pain Department of the Second People's Hospital of Zhangjiagang City, Jiangsu Province. The patients were recommended to participate in the trial by the department physicians. The recruitment was started in October 2021 and completed by the end of June 2024. In all, 413 cases were evaluated for eligibility. After applying the inclusion and exclusion criteria defined for this study, 184 subjects with rotator cuff injuries were recruited in this study, with 92 cases in each group. While the control group only received the ultrasound-guided hypertonic dextrose injection treatment, the experimental group received the same injection in combination with TCM fumigation treatment. All subjects were informed of the general steps of the treatment procedures, as well as the curative effects and possible risks associated with the treatment methods. After the subjects agreed to participate in this study and signed the informed consent form for the clinical trial, they were randomly assigned to either of the two groups.

#### 2.3.1 Diagnostic criteria

The diagnosis of rotator cuff injury was made according to the diagnostic criteria defined in the 2019 Clinical Practice Guideline for the Management of Rotator Cuff Injury by the American Academy of Orthopedic Surgeons (AAOS) (14).

#### 2.3.2 Inclusion criteria

Subjects were included in this study if (1) the diagnosis of rotator cuff injury was clearly established; (2) the course of the disease was 24 months or less; and (3) the age of the patient was between 20 and 85 years.

#### 2.3.3 Exclusion criteria

Patients meeting any of the following criteria were excluded from this study:(1) presence of other diseases such as soft tissue injuries of the neck, shoulders, back, or upper limbs; entrapment of peripheral nerves and blood vessels in the upper limbs; or fractures that may affect the results of this study; (2) coexisting severe disorders of the heart, lungs, liver, or kidney function; tumors; diseases of the hematopoietic system; mental disorders; and pregnancy; (3) acute episodes of chronic diseases such as hypertension, diabetes, and chronic nephritis; (4) use of drugs or other treatment methods that may affect the evaluation of the curative effect before participation in the trial that may affect the evaluation of curative effect; (5) positive allergic reaction to the test drugs; and (6) refusal to participate in the trial or sign the informed consent form for the clinical trial.

#### 2.3.4 Attrition criteria

(1) Subjects who failed to complete the required number of treatment sessions during the trial; (2) Subjects lost to follow-up after treatment; (3) Subjects who experienced serious adverse events during the trial; (4) Subjects who voluntarily withdrew from the trial during the study.

### 2.4 Randomization

A physician at our hospital who did not participate in the study used a computerized random number generator to generate a random sequence of the numbers 1 to 184, which was then placed in a sealed, opaque, continuously coded envelope in triplicate. One copy each was maintained with the study director, treating physician, and statistician. Before enrollment, all three simultaneously personally opened the envelopes after obtaining them in the coded order. Subjects were allocated to the experimental or control group based on their assigned randomization sequence number: odd numbers indicated allocation to the experimental group, and even numbers to the control group.

### 2.5 Treatment Methods

#### 2.5.1 Experimental group

(1) Ultrasound-guided hypertonic dextrose injection: A 5-ml single-use sterile syringe was used to draw 2.5 ml of 50% dextrose injection (China Otsuka Pharmaceutical Co., Ltd.) and 2.5 ml of sterile water for injection (Shiyao Yinhu Pharmaceutical Co., Ltd.) to prepare 5 ml of 25% hypertonic dextrose injection for standby. The PHILIPS EPIQ7C vertical ultrasound (Royal Philips of the Netherlands) device was used for ultrasound guidance, with a 5–12 MHz high-frequency linear array probe set at the musculoskeletal scanning mode. The depth of the scan was chosen appropriately and gain was adjusted according to the subject's body build. The coupling agent was applied on the probe, and a single-use sterile glove was placed, tied up tightly, and kept ready after disinfection.

**Figure 1:**
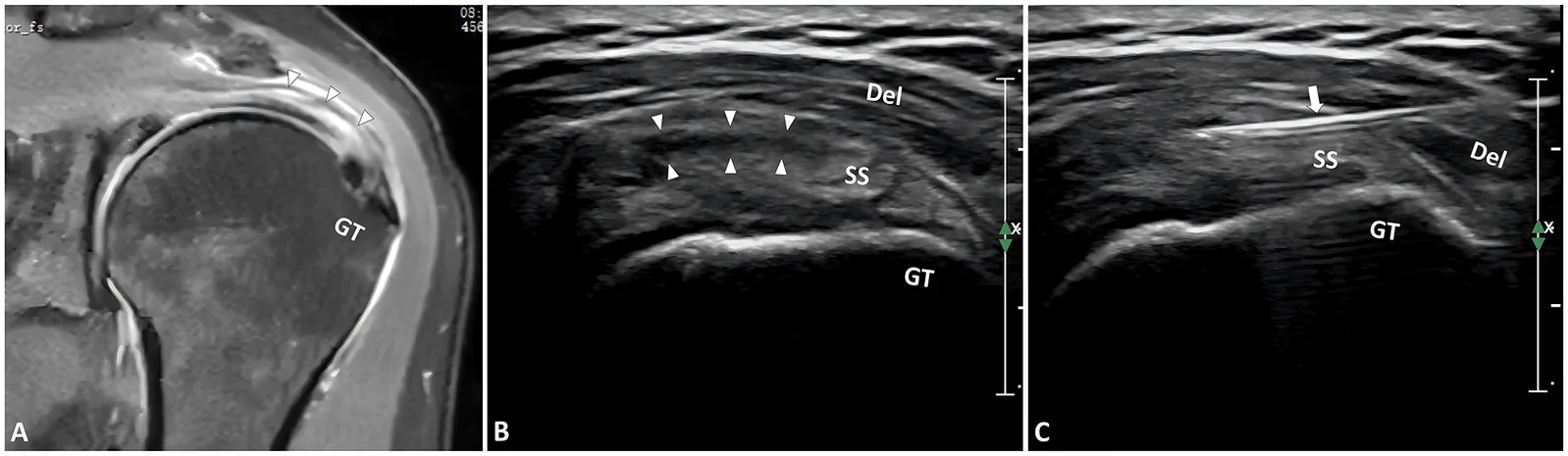
Hypertonic dextrose injection at the site of supraspinatus tendon tear under ultrasound guidance. **(A)** An intratendinous tear of the supraspinatus tendon is shown on the oblique coronal T2W1 MR image of the shoulder joint (short arrow). **(B)** In the long-axis scan of the supraspinatus tendon by ultrasound, a long strip-shaped hypoechoic area (short arrow) appears within the tendon, indicating an intratendinous tear of the supraspinatus tendon. **(C)** Under ultrasound guidance, the injection needle (long arrow) reaches the tear site of the supraspinatus tendon. GT, greater tubercle of humerus; SS, supraspinatus tendon; Del, deltoid muscle.

The subject was placed in the supine position, with the affected shoulder fully exposed, and the position was adjusted according to the tendon to be scanned. For example, for the supraspinatus tendon, the affected shoulder joint was internally rotated, the elbow flexed, the upper limb placed behind the back, with the palm placed upward on the upper edge of the iliac crest. The affected shoulder was then routinely disinfected with iodophor and covered with a sterile drape. The probe marker was pointed toward the acromion, and the supraspinatus tendon was scanned in the long-axis plane. Ultrasound guidance was used in combination with magnetic resonance images to assess the location and degree of tendon injury and determine the direction and angle of needle insertion. Using the in-plane needle insertion technique, the needle was slowly introduced from the outer-lower to the inner-upper direction under ultrasound guidance. Once the needle tip reached the site of tendon injury and no blood was drawn, the hypertonic dextrose solution was slowly injected (Figure 1). After the needle was withdrawn, pressure was applied to the site of needle puncture to arrest bleeding, and an infusion patch was used for coverage. A single treatment course comprised 3–5 such injections administered once a week.

**Figure 2:**
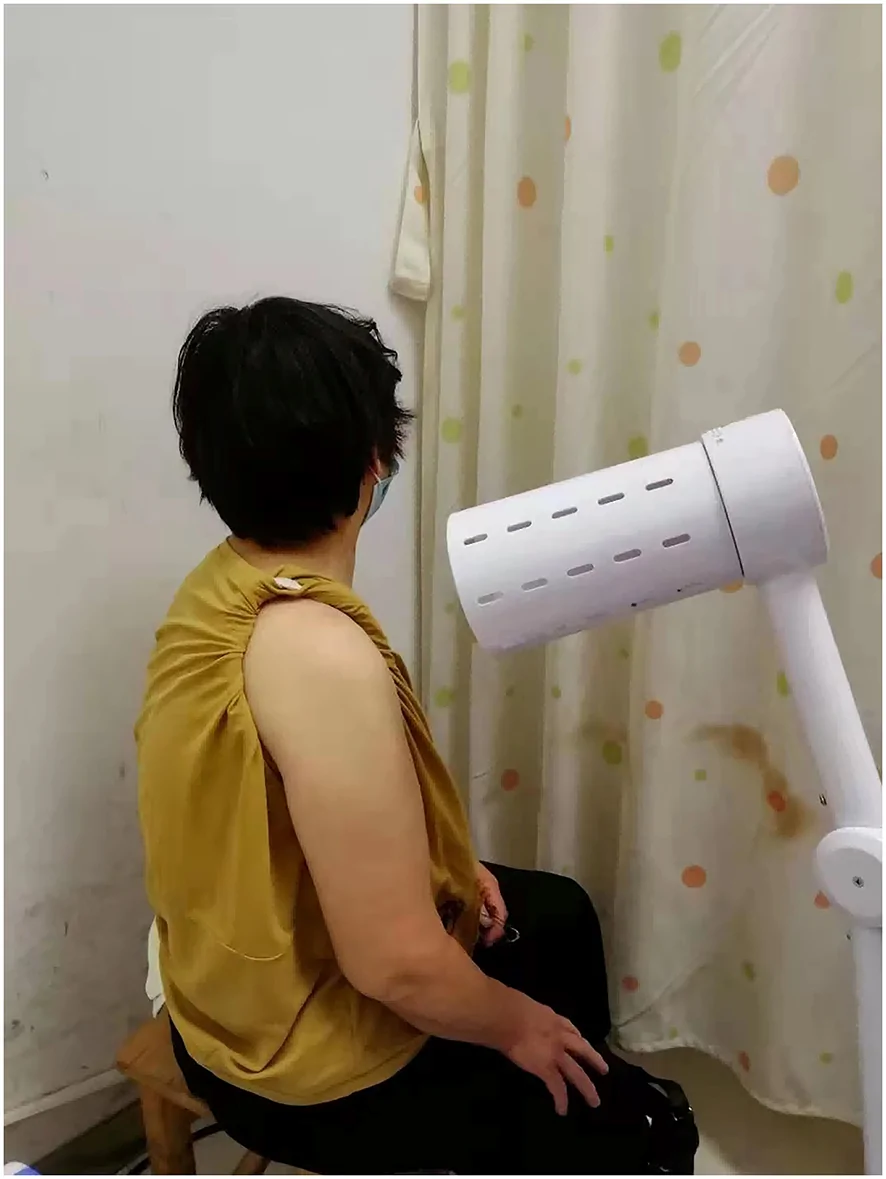
Subjects undergoing traditional Chinese medicine fumigation.

(2) TCM fumigation: the experimental group was started on TCM fumigation treatment 1–2 days after administration of each injection. TCM fumigation was performed using the Relaxing Tendons and Activating Collaterals Lotion, an empirical prescription developed by the late TCM orthopedics expert from our hospital, Teacher Ma Yunliang. The composition of the prescription is as follows: 20 g each of Radix Clematidis, Chuanxiong Rhizoma, Atractylodis Rhizoma, Erythrina Bark, Cyathulae Radix, and Chaenomelis Fructus; 15 g each of Angelicae Pubescentis Radix, Notopterygii Rhizoma Et Radix, Taxilli Herba, Common Clubmoss Herb, Herb of Tuberculate Speranskia, Saposhnikoviae Radix; 10 g each of Safflower, Frankincense, Commiphora Myrrha, Radix Aconiti Kusnezoffii Preparata, Radix Aconiti Preparata; and 100 mL of high-proof liquor. All the medicinal ingredients were placed in a pot, followed by the addition of 3,000 ml of water, and allowed to soak for 30 min. Once boiling was reached at a high flame, the preparation was allowed to simmer for 30 min at a low flame. Then, the medicinal residues were passed through a strainer, and the filtered TCM liquid was added to the fumigation treatment machine (Xiangyu brand HYZ-IE, Henan Xiangyu Medical Equipment Co., Ltd.). The subject was required to assume the sitting position, with the affected shoulder exposed. The fumigation treatment machine was then turned on, the temperature adjusted, and the nozzle aimed at the area around the injured tendon for fumigation. The nozzle was placed about 15–20 cm away from the affected area, and each fumigation session lasted about 30 min (Figure 2). The fumigation treatment was carried out twice a week, and one course of treatment lasted 3–5 weeks, depending on the number of injections.

#### 2.5.2 Control group

The control group only received ultrasound-guided hypertonic dextrose injection, with the treatment method and course being the same as those of the experimental group.

During the study period, subjects were instructed to refrain from other interventions that could potentially confound therapeutic outcomes, including acupuncture, massage, physical therapy, topical plaster application, steroid injection, and oral anti-inflammatory/analgesic agents, until the completion of the follow-up period. Additionally, they were advised to avoid strenuous shoulder joint movements.

### 2.6 Observation indicators

The primary outcome indicator of this study was the CMS score (at a total score of 100 points; the higher the score, the better the condition). The secondary outcome indicators included the Modified University of California at Los Angeles Shoulder Rating Scale (modified UCLA) (15) (total score: 0–35 points; the higher the score, the better the condition), the Visual Analog Scale (VAS) score (total score: 0–10 points; the higher the score, the more severe the pain), and the clinical efficacy (rate of cure and marked effectiveness; total effectiveness rate). The clinical efficacy was assessed on the basis of the rate of post-treatment improvement in the CMS score. Improvement rate was determined as follows: improvement rate = [(total score after treatment-total score before treatment)/(100-total score before treatment)] × 100%. On the basis of the improvement rates, the outcomes were classified as cure, markedly effective, effective, or ineffective. Cure was defined as ≥ 90% improvement in the score after treatment. Marked effectiveness was defined by a ≥60% and <90% improvement rate in the post-treatment score. Effectiveness was defined by a ≥30% to <60% improvement in the post-treatment score. Ineffectiveness of the treatment was defined by <30% improvement in the post-treatment score. The rate of cure and marked effectiveness was calculated as follows: [(number of cases of cure + number of cases of marked effect)/total number of cases] × 100%. The total rate of effectiveness was determined using the formula [(number of cases of cure + number of cases of marked effect + number of cases of effective)/total number of cases] × 100%.

With respect to the safety indicators, adverse reactions were defined as vasovagal response during or after treatment, local hematoma at the injection site, infection, drug allergy, skin scald, recurrence or exacerbation of pre-existing diseases.

### 2.7 Protocol deviation

The secondary outcome measure registered with the Chinese Clinical Trial Registry (ChiCTR)—the Short-Form McGill Pain Questionnaire (SF-MPQ)—was intended to assess subjects' pain intensity. To enhance subjects' comprehension of their pain ratings, we revised this outcome measure to the VAS score prior to subject recruitment. This protocol amendment was reported to the sponsor and approved by the institutional review board (IRB). The change constitutes a minor protocol deviation, as it does not materially affect subjects safety, rights, or welfare, nor does it compromise the scientific validity of the trial data.

### 2.8 Follow-up protocol for subjects

Subjects were followed up mainly as outpatients, with telephonic interviews, home visits, and online surveys conducted as required. General demographic and clinical data of the subjects were collected before enrollment. A single-blind method was used for the follow-up personnel to collect data on the subject's CMS score, modified UCLA score, and VAS score before enrollment and at 2 months and 6 months after the end of the treatment course. Clinical efficacy was evaluated 6 months after the completion of the treatment course. Adverse reactions of the subjects were recorded during after the treatment and each follow-up.

### 2.9 Statistical analysis

The data were statistically analyzed by statisticians using the blind method. The data of the two groups were analyzed using the intention-to-treat (ITT) analysis set. To evaluate the robustness of the primary study conclusions, a prespecified sensitivity analysis was conducted using the per-protocol (PP) population. Subjects were excluded from the PP population if they met any of the prespecified attrition criteria outlined in the statistical analysis plan. The safety of the clinical trial was evaluated using the safety-analysis-set (SS). Statistical analysis was performed using the SPSS 27.0 software. Given that the missing data followed a missing at random (MAR) mechanism, we employed multiple imputation by chained equations (MICE) to address the missing values. Categorical variables were expressed as the number of cases (percentage) [*n* (%)], and intergroup comparisons of the rates were made using the χ^2^ test. The phi coefficient was used as the corresponding effect size to reflect the strength of association between categorical variables: a phi value <0.1 indicates a negligible effect; values between 0.1 and 0.3, a small effect; values between 0.3 and 0.5, a moderate effect; and values > 0.5, a large effect. Continuous variables with normal distribution were expressed as mean ± standard deviation (M ± SD). Paired-sample *t*-test was used for intragroup comparison, and independent-sample *t*-test was used for intergroup comparison. The standardized effect size for the difference in means between the two groups was calculated using Cohen's *d*. Effect sizes were interpreted according to Cohen's conventional thresholds: small (*d* = 0.20), medium (*d* = 0.50), and large (*d* = 0.80). Continuous variables with non-normal distribution were expressed as median (first quartile, third quartile) [*M* (*P*_25_, *P*_75_)]. The Wilcoxon signed-rank test was used for intragroup comparison, while the Mann-Whitney U test was used for intergroup comparison. The standardized effect size was quantified using Pearson's correlation coefficient (*r*). The strength of the correlation was interpreted according to Cohen's criteria: small (*r* = 0.10), medium (*r* = 0.30), and large (*r* = 0.50). The initial threshold for statistical significance was set at *P* < 0.05. The adjusted significance level following multiple comparisons correction via the Bonferroni method was set as follows: α′ = 0.05/3 = 0.017, *P* < 0.017 was considered indicative of a statistically significant difference.

## 3 Results

### 3.1 Intergroup comparison of baseline data, inclusion status, and dropout status

**Figure 3:**
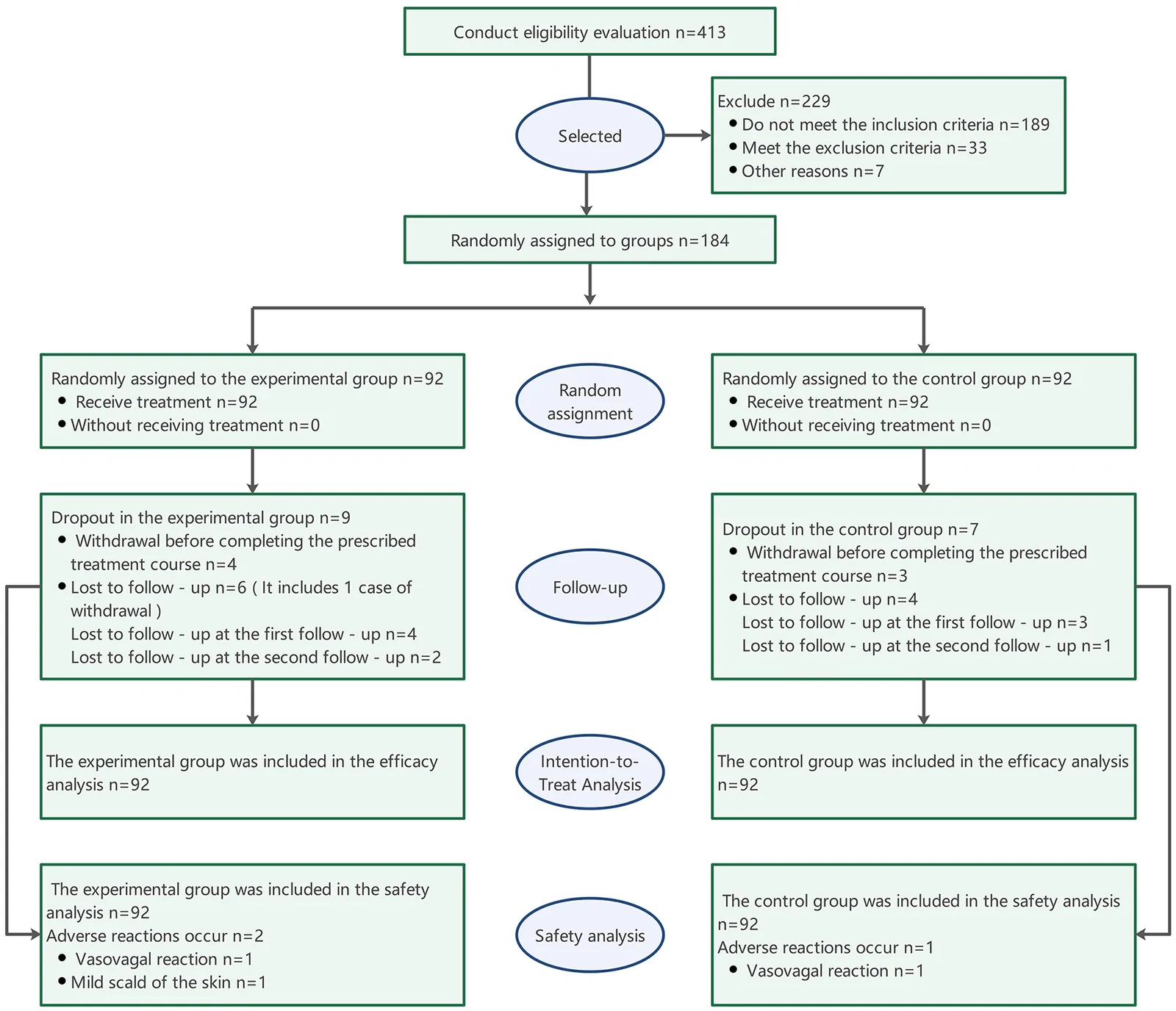
Research flow chart.

The baseline data of the two groups were compared (Table 1). During the study period, nine subjects dropped out of the experimental group (dropout rate: 9.8%), while seven subjects dropped out of the control group (dropout rate of 7.6%). The difference in the dropout rates in the two groups was not statistically significant [RD = 0.02, 95% CI (−0.06, 0.11), ϕ = 0.04, χ^2^ = 0.27, *P* = 0.601], with a negligible effect size. Among the dropout cases, 4 in the experimental group and three in the control group withdrew due to failure to complete the treatment course as required. Six subjects in the experimental group were lost to follow-up, four at the first follow-up (including one who withdrew from the trial) and two at the second follow-up. Four subjects in the control group were lost to follow-up, three at the first follow-up and one at the second follow-up (Figure 3).

**Table 1:** Comparison of demographic and clinical characteristics of subjects in two groups.

Baseline name	Experimental group (*n* = 92)	Control group (*n* = 92)	RD or MD (95% CI)	Effect size	*P*-value
Sex *n* (%)					
Male	31 (33.7)	25 (27.2)	0.07 [−0.07, 0.20]	ϕ = 0.07	0.336
Female	61 (66.3)	67 (72.8)			
Age (years) mean ± SD	61.0 ± 12.0	60.8 ± 9.9	0.17 [−3.02, 3.37]	Cohen's *d* = 0.01	0.915
Course of disease (month) M (*P*_25_, *P*_75_)	2.0 (1.0, 5.0)	2.0 (1.0, 5.0)	0.00 [0.00, 1.00]	*r* = 0.04	0.631
Affected side *n* (%)					
Right	54(58.7)	57 (62.0)	0.03 [−0.11, 0.17]	ϕ = 0.03	0.651
Left	38 (41.3)	35 (38.0)			
Etiology *n* (%)					
Exertion injury	84 (91.3)	82 (89.1)	0.02 [−0.07, 0.11]	ϕ = 0.04	0.620
Trauma	8 (8.7)	10 (10.9)			
Grading (Ellman) *n* (%)					
Degree I (depth <3 mm)	47 (51.1)	44 (47.8)	0.03 [−0.11, 0.17]	ϕ = 0.03	0.658
Degree II (depth 3 6 mm)	33 (35.9.)	39 (42.4)	0.07 [−0.08, 0.20]	ϕ = 0.07	0.365
Degree III (depth >6 mm)	12 (13.0)	9 (9.8)	0.03 [−0.06, 0.13]	ϕ = 0.05	0.487
Treatment cycle *n* (%)					
<3 weeks	4 (4.4)	3 (3.3)	0.01 [−0.05, 0.08]	ϕ = 0.03	0.700
3 weeks	30 (32.6)	33 (35.9)	0.03 [−0.10, 0.17]	ϕ = 0.03	0.641
4 weeks	33 (35.9)	28 (30.4)	0.05 [−0.08, 0.19]	ϕ = 0.06	0.434
5 weeks	25 (27.1)	28 (30.4)	0.03 [−0.10, 0.16]	ϕ = 0.04	0.625

*n*, number of cases; mean ± SD, mean ± standard deviation; RD, rate difference; MD, mean difference or median difference; M (*P*_25_, *P*_75_), median (the first quartile, the third quartile); 95% CI, 95% confidence interval.

### 3.2 Comparison of CMS scores before and after treatment in the two groups

**Figure 4:**
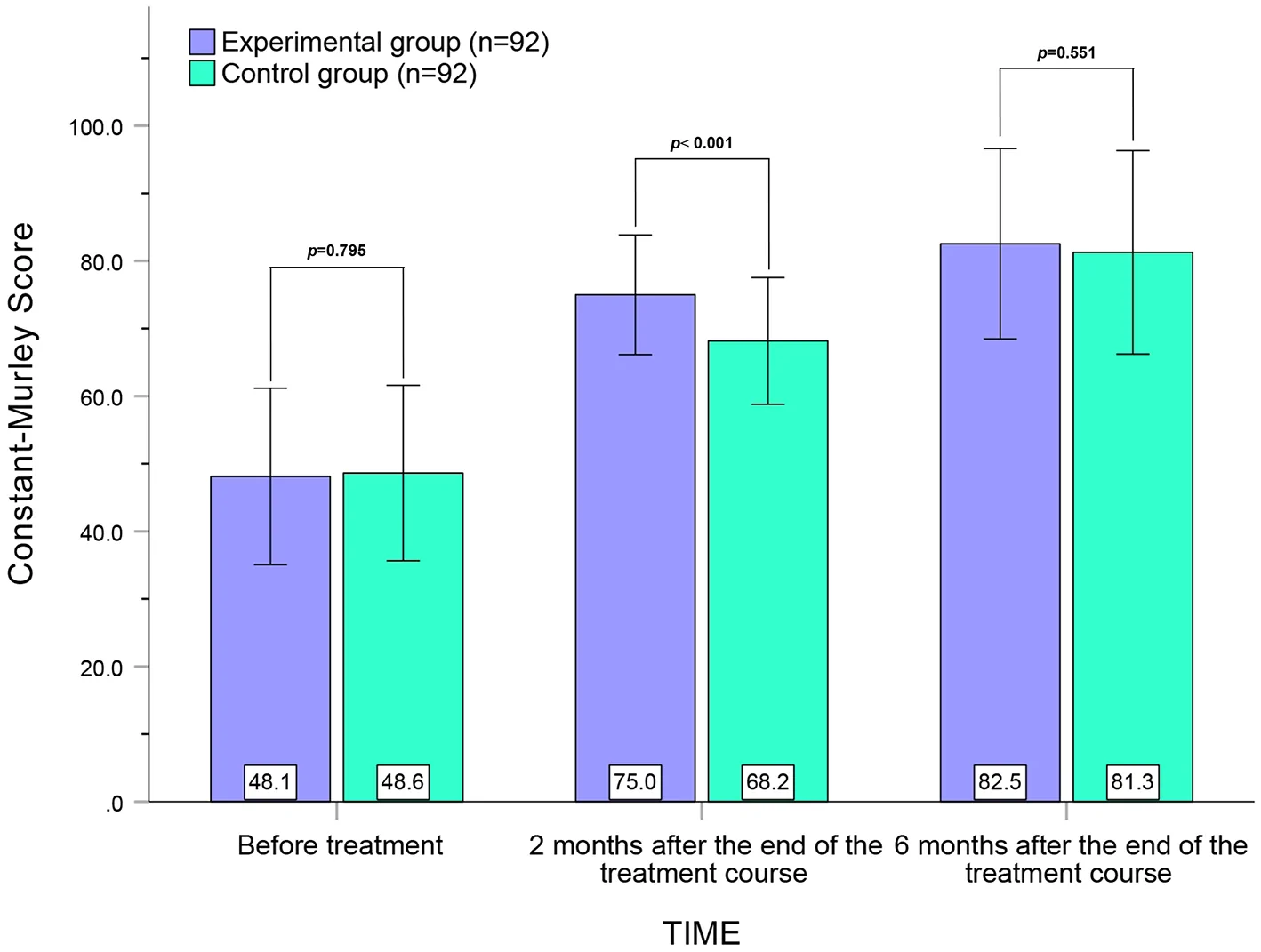
Comparison of Constant-Murley Shoulder scores of the shoulder joint before and after treatment between two groups of subjects with rotator cuff injuries.

The pretreatment CMS scores in the experimental group and control groups were 48.1 ± 13.1 points and 48.6 ± 13.0 points, respectively, indicating no statistically significant differences between the two groups [MD = −0.50, 95% CI (−4.27, 3.29), Cohen's *d* = 0.04, *t* = −0.26, *P* = 0.795], with a negligible effect size. Two months after the completion of the treatment course, the CMS score showed a significant improvement from the pretreatment scores both in the experimental group 75.0 ± 8.8 points; [MD = 26.87, 95% CI (23.93, 29.81), Cohen's *d* = 3.05, *t* = 18.17, *P* < 0.001] and in the control group 68.2 ± 9.4 points; [MD = 19.54, 95% CI (16.74, 22.34), Cohen's *d* = 2.08, *t* = 13.84, *P* < 0.001], both groups exhibited a large effect size. However, compared with the control group, the improvement in scores of the experimental group was greater, and the difference was statistically significant [MD = 6.83, 95% CI (4.18, 9.48), Cohen's *d* = 0.75, *t* = 5.09, *P* < 0.001], indicating a medium effect size. Thus, it can be concluded that the adjunctive application of TCM fumigation to the conventional treatment regimen significantly enhances shoulder joint function in subjects within 2 months of completing the treatment. Six months after the completion of the treatment course, the CMS score in the experimental group was 82.5±14.1 points, indicating a significant improvement from the pretreatment score [MD = 34.42, 95% CI (30.77, 38.08), Cohen's *d* = 2.44, *t* = 18.72, *P* < 0.001]. Similarly, the score in the control group was 81.3 ± 15.1 points, which showed a significant improvement from the pretreatment score [MD = 32.64, 95% CI (28.75, 36.54), Cohen's *d* = 2.16, *t* = 16.65, *P* < 0.001], both groups exhibited a large effect size. However, the differences between the scores in the two groups were not statistically significant [MD = 1.28, 95% CI (−2.96, 5.52), Cohen's *d* = 0.09, *t* = 0.60, *P* = 0.551], with a negligible effect size (Figure 4). This suggests that the additional therapeutic effects conferred by TCM fumigation tend to gradually diminish over time.

### 3.3 Comparison of the pre- and post-treatment VAS scores in the two groups

**Figure 5:**
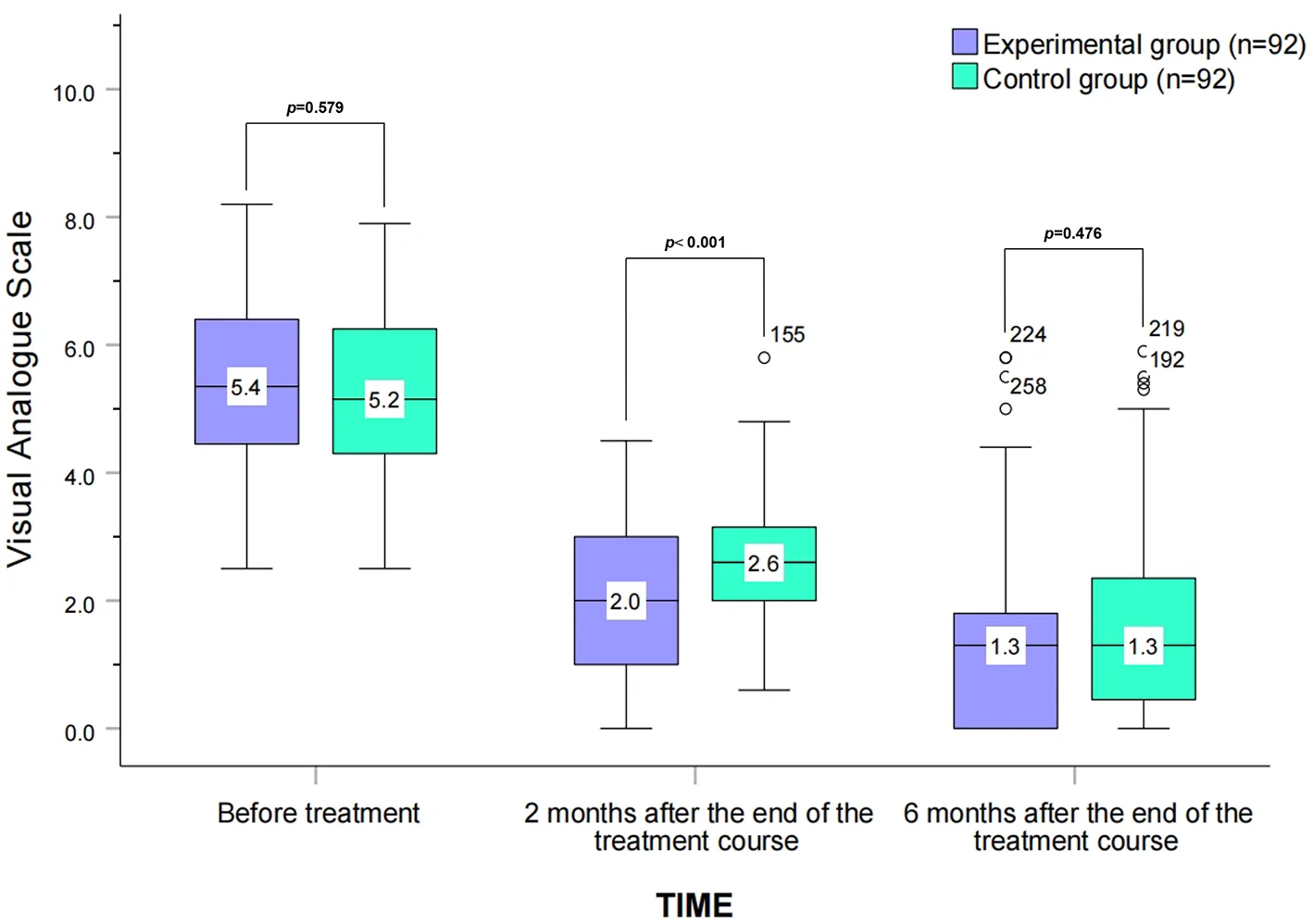
Comparison of Visual Analog Scale score of the shoulder joint before and after treatment between the two groups of subjects with rotator cuff injuries.

The pretreatment VAS scores in the experimental group and control group were 5.4 (4.4, 6.4) and 5.2 (4.3, 6.3), respectively, indicating no statistically significant intergroup differences in the scores [MD = 0.10, 95% CI (−0.30, 0.50), *r* = 0.04, *Z* = −0.56, *P* = 0.579], with a negligible effect size. Two months after the completion of the treatment course, the VAS score in the experimental group was significantly lower than the pretreatment score, at 2.0 (1.0, 3.0) points [MD = −3.30, 95% CI (−3.65, −3.00), *r* = 0.87, *Z* = −8.33, *P* < 0.001], while that in the control group also significantly lower than the pretreatment score, at 2.6 (2.0, 3.2) [MD = −2.45, 95% CI (−2.80, −2.15), *r* = 0.87, *Z* = −8.33, *P* < 0.001], both groups exhibited a large effect size. However, the reduction in the scores in the experimental group was significantly greater than that in the control group [MD = −0.70, 95% CI (−1.00, −0.30), *r* = 0.29, *Z* = −3.92, *P* < 0.001], indicating a medium effect size. Therefore, it can be concluded that the adjunctive application of TCM fumigation therapy to the conventional treatment regimen significantly alleviates shoulder joint pain in subjects within 2 months of completing treatment. Similarly, 6 months after the completion of the treatment course, the VAS scores in the experimental group and control group were 1.3 (0.0, 1.8) points 1.3 (0.4, 2.5) points, respectively, which were significantly lower than the pretreatment scores in the respective groups [MD = −3.95, 95% CI (−4.40, −3.50), *r* = 0.87, *Z* = −8.31, *P* < 0.001 and MD = −3.55, 95% CI (−4.05, −3.10), *r* = 0.87, *Z* = −8.29, *P* < 0.001, respectively], both groups exhibited a large effect size. However, the differences in the scores in the two groups were not statistically significant [MD = 0.00, 95% CI (−0.50, 0.10), *r* = 0.06, *Z* = −0.83, *P* = 0.476], with a negligible effect size (Figure 5).

### 3.4 Comparison of modified UCLA scores before and after treatment in the two groups

**Figure 6:**
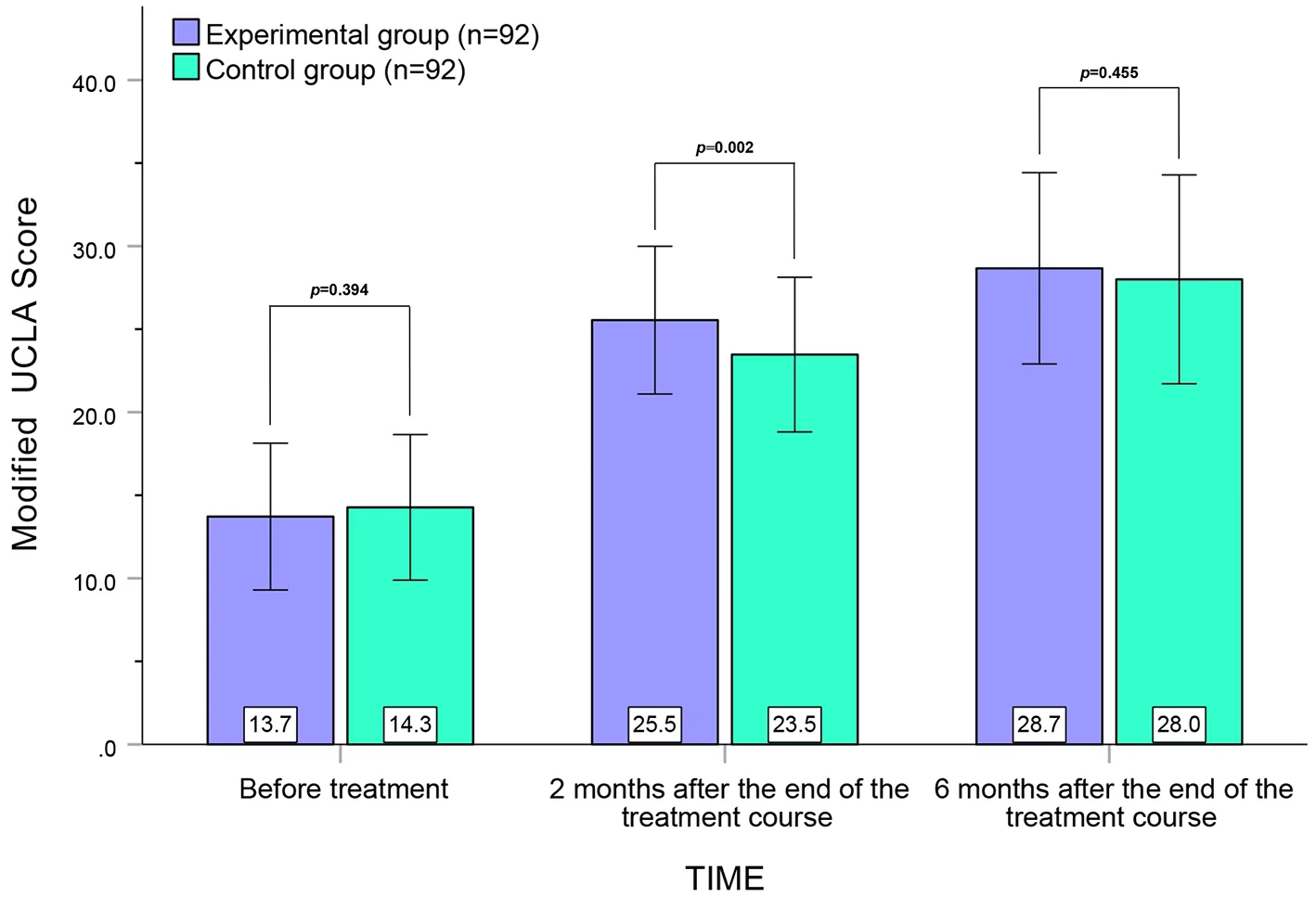
Comparison of Modified University of California at Los Angeles Shoulder Rating Scale scores before and after treatment between two groups of subjects with rotator cuff injuries.

The pretreatment modified UCLA scale score was 13.7 ± 4.4 in the experimental group and 14.3 ± 4.4 in the control group, indicating no statistically significant difference between the two groups [MD = −0.55, 95% CI (−1.84, 0.73), Cohen's *d* = 0.13, *t* = −0.85, *P* = 0.394], indicating the presence of a small effect. Two months after the completion of the treatment course, the modified UCLA scale score in the experimental group was 25.5±4.4 points, which was significantly higher than that before treatment [MD = 11.83, 95% CI (10.57, 13.08), Cohen's *d* = 2.69, *t* = 18.71, *P* < 0.001]; similarly, the score in the control group was 23.5±4.7 points, which was significantly higher than that before treatment [MD = 9.20, 95% CI (8.02, 10.38), Cohen's *d* = 1.96, *t* = 15.51, *P* < 0.001], both groups exhibited a large effect size. Compared with the control group, the score improvement in the experimental group was greater, and the difference was statistically significant [MD = 2.07, 95% CI (0.75, 3.40), Cohen's *d* = 0.46, *t* = 3.09, *P* = 0.002], small-to-moderate effect size. Thus, it can be concluded that the adjunctive application of TCM fumigation therapy to the conventional treatment regimen significantly enhances shoulder joint range of motion, muscle strength, and subject satisfaction within 2 months following the completion of the treatment course. Six months after the completion of the treatment course, the modified UCLA scale score in the experimental group was 28.7 ± 5.8 points, which was significantly higher than that before treatment [MD = 14.95, 95% CI (13.54, 16.36), Cohen's *d* = 2.58, *t* = 21.05, *P* < 0.001]. The score in the control group was 28.0 ± 6.3 points, which was significantly higher than the pretreatment score [MD = 13.73, 95% CI (12.18, 15.28), Cohen's *d* = 2.18, *t* = 17.62, *P* < 0.001], both groups exhibited a large effect size. However, there was no statistically significant difference in the scores of the two groups [MD = 0.67, 95% CI (−1.09, 2.42), Cohen's *d* = 0.11, *t* = 0.75, *P* = 0.455], indicating the presence of a small effect (Figure 6).

### 3.5 Comparison of curative effects between the two groups of subjects 6 months after the end of the treatment course.

Six months after the completion of the treatment course, the rate of cure and marked effect in the experimental group was 68.5%, while that in the control group was 64.1%. The difference between the rates in the two groups was not statistically significant [RD = 0.04, 95% CI (−0.09, 0.18), ϕ = 0.05, χ^2^ = 0.39, *P* = 0.533], with a negligible effect size. The total rate of treatment effectiveness in the experimental group was 82.6%, while that in the control group was 81.5%, indicating no statistically significant difference between the two groups [RD = 0.01, 95% CI (−0.10, 0.12), ϕ = 0.01, χ^2^ = 0.04, *P* = 0.848], with a negligible effect size (Table 2).

**Table 2:** Comparison of clinical efficacy between the two groups of subjects with rotator cuff tears at 6 months post-treatment.

The clinical curative effect	Experimental group (*n* = 92)	Control group (*n* = 92)	RD (95% CI)	*P*-value
Cure *n* (%)	20 (21.8)	19 (20.6)	-	-
Marked effect *n* (%)	43 (46.7)	40 (43.5)	-	-
Effective *n* (%)	13 (14.1)	16 (17.4)	-	-
Invalid *n* (%)	16 (17.4)	17 (18.5)	-	-
Cure and marked effective rate (%)	68.5	64.1	0.04 [−0.09, 0.18]	0.533
Total effective rate (%)	82.6	81.5	0.01 [−0.10, 0.12]	0.848

*n*, number of cases; RD, rate difference; 95% CI, 95% confidence interval.

### 3.6 Comparison of safety between the two groups of subjects

In the experimental group, one subject had a mild vasovagal reaction at the end of the treatment, while another had mild scalding over a small area during the fumigation procedure. Thus, the incidence of adverse reactions in the experimental group was 2.2%. In the control group, one subject developed a mild vasovagal reaction during the treatment, making the incidence of adverse reactions 1.1%. The difference between the incidence of adverse reactions in the two groups was not statistically significant [RD = 0.011, 95% CI (−0.04, 0.07), ϕ = 0.04, χ^2^ = 0.34, *P* = 0.560], with a negligible effect size. All subjects achieved complete recovery from the adverse reactions after symptomatic treatment; there were no instances of adverse consequences in any of the subjects.

### 3.7 Sensitivity robustness analysis comparing ITT and PP populations

The ITT analysis population comprised 92 subjects in the experimental group and 92 in the control group. The PP analysis population excluded 16 participants with major protocol deviations, resulting in a final analytic sample of 168 subjects-−83 in the experimental group and 85 in the control group. The primary reason for exclusion was subject attrition. In the ITT population, the between-group difference in the primary outcome—CMS score—was statistically significant at 2 months [MD = 6.83, 95% CI (4.18, 9.48), Cohen's *d* = 0.75, *t* = 5.09, *P* < 0.001], but no statistically significant at 6 months [MD = 1.28; 95% CI (−2.96, 5.52); Cohen's *d* = 0.09; *t* = 0.60; *P* = 0.551]. In the PP population, the between-group difference in the primary outcome—CMS score—was statistically significant at 2 months [MD = 7.05, 95% CI (4.28, 9.82), Cohen's *d* = 0.77, *t* = 5.03, *P* < 0.001], but no statistically significant at 6 months [MD = 1.41; 95% CI (−3.16, 5.99); Cohen's *d* = 0.09; *t* = 0.61; *P* = 0.543]. Effect direction, 95% confidence intervals, and statistical significance were highly consistent between the ITT and PP populations, with only trivial differences in point estimates. These findings demonstrate the robustness and reliability of the primary conclusions, confirming that results are not meaningfully altered by protocol deviations.

## 4 Discussion

The tendons that form the rotator cuff inherently have poor blood supply and limited reparative capacity, making them highly susceptible to injury. Notably, the supraspinatus tendon at its insertion onto the greater tubercle of the humerus has the most compromised vascularity, which makes post-injury healing difficult. Rotator cuff injuries are most often associated with age-related chronic degenerative lesions, followed by acute trauma (16). According to the depth of tendon involvement, rotator cuff injuries can be classified into full-thickness tears and partial-thickness tears. Full-thickness tears are further subclassified by size: small tears (less than 1 cm), moderate tears (1–3 cm), large tears (3–5 cm), and massive tears (greater than 5 cm). On the basis of the anatomic site of tears, rotator cuff injuries may be categorized as bursal-side tears, articular-side tears, and intratendinous tears (17).

Injections administered for rotator cuff injuries in clinical practice commonly include platelet-rich plasma, corticosteroids, and hypertonic dextrose. Corticosteroids afford pain relief and short-term improvement in function, while platelet-rich plasma and hypertonic dextrose can exert long-term curative effects (18, 19). On the other hand, hypertonic dextrose injection is safe with no side effects, technically simple, and less expensive, while also yielding long-term curative effects; therefore, it is a better alternative to the others for patients with rotator cuff injuries (20).

After the hypertonic dextrose solution is injected into the lesion site, it can trigger a local inflammatory reaction, resulting in the excess production of endogenous growth factors with increased metabolic activity of chondrocytes, collagen deposition, and chondrocyte proliferation (10, 21). New collagen is used for the production of articular cartilage, tendons, ligaments and to strengthen muscles. Currently, 10%−25% dextrose solution is commonly used in clinical practice. However, high-concentration dextrose solutions have been shown to improve tendon and osteoarticular diseases to a greater extent than low-concentration dextrose solutions (22, 23). In this study, we used 25% hypertonic dextrose injection. Significant improvement was seen in shoulder joint function in both the experimental group and the control group compared to the pretreatment status both at 2 months and at 6 months after the completion of the treatment course; a continued decline in the pain scores was observed.

Administration of the injection under ultrasound guidance allows for the precise identification of the lesion site and real-time tracking of the needle tip, without the need for radiation exposure. This is supported by the latest guidelines of the European Society of Musculoskeletal Radiology (ESSR), which suggest that most ultrasound-guided interventional procedures for the shoulder joint are more accurate and effective than blind procedures (24). Adamson et al. (25) compared the effectiveness of ultrasound-guided and bony landmark–guided corticosteroid injections for pain related to rotator cuff injuries and found that ultrasound-guided injection affords better outcomes and rarely causes adverse reactions. An earlier study investigating ultrasound-guided hypertonic dextrose injection in the treatment of refractory rotator cuff injuries revealed that the VAS score, Shoulder Pain and Disability Index (SPADI), and Western Ontario Rotator Cuff Index (WORC) all improved significantly from the pretreatment levels, with an excellent rate of improvement (92.9% of cases) at 1-year follow-up (26). In this study, we used ultrasound to first locate the lesion site and plan the puncture path, which enabled the needle tip to reach the lesion site quickly and accurately, shortened the procedure, and reduced the damage and pain caused by repeated adjustment of the puncture direction to the subjects, thereby improving the efficacy and safety.

Some patients may develop an exacerbation of pain within 1 to 3 days of the administration of the hypertonic dextrose injection into the rotator cuff due to an inflammatory response (27); this may make patients doubt the treatment effect. Therefore, we used TCM that has the effects of relaxing tendons and activating collaterals, activating blood circulation and relieving pain, and strengthening tendons and bones to fumigate the affected shoulder within 1 or 2 days of the administration of hypertonic dextrose injection. Our results showed that the CMS scores and modified UCLA scores of the subjects at the end of 2 months of the treatment achieved with the combined application of TCM fumigation treatment were significantly higher and the pain scores significantly less than those of the group treated with ultrasound-guided hypertonic dextrose injection alone.

TCM fumigation employs thermally generated vapor to induce cutaneous pore dilation, vasodilation, and enhanced epidermal permeability—thereby promoting transdermal delivery of bioactive constituents (28, 29). Moreover, it ameliorates microcirculatory dysfunction, modulates autonomic and somatosensory neural activity, and elevates the nociceptive threshold, collectively fostering a regenerative microenvironment for neural and musculoskeletal tissues (30). Pharmacological evidence indicates that alkaloids and flavonoids derived from core formula components—including Chuanxiong Rhizoma, Safflower, and Radix Clematidis—exert vasodilatory effects and augment regional perfusion in shoulder tissues, while accelerating the clearance of pro-inflammatory metabolites. Volatile oil constituents from Angelicae Pubescentis Radix, Notopterygii Rhizoma Et Radix, and Chaenomelis Fructus suppress cyclooxygenase-mediated prostaglandin synthesis and attenuate peripheral nociceptor sensitization. Resinous compounds from Frankincense and Commiphora Myrrha, alongside diterpenoid alkaloids from processed Radix Aconiti Preparata and Radix Aconiti Kusnezoffii Preparata, contribute synergistically to analgesia. Furthermore, Common Clubmoss Herb and Herb of Tuberculate Speranskia enhance collagenolytic activity via upregulation of matrix metalloproteinase-1 (MMP-1) and tissue inhibitor of metalloproteinases-1 (TIMP-1) balance, thereby improving tendon and joint capsule extensibility and restoring functional range of motion (31). Mechanistically, TCM fumigation downregulates mRNA expression of pro-inflammatory mediators—including interleukin-6 (IL-6), tumor necrosis factor-alpha (TNF-α), and nuclear factor-kappa B subunit p65 (NF-κB p65)—while concurrently enhancing transcription of the endogenous inhibitor IκBα in inflamed periarticular tissues, resulting in attenuated local inflammation, reduced edema, sustained analgesia, and improved health-related quality of life (32).

TCM fumigation is a commonly used approach for the treatment of rotator cuff injuries in TCM; similar to other external TCM therapies such as acupuncture, floating needle therapy, and massage, fumigation has been shown to afford good clinical results in rotator cuff injuries (11). Several recent studies have confirmed that TCM fumigation can effectively improve the symptoms of knee osteoarthritis, post-operative hip dysplasia in children, and acute ankle sprains, thereby promoting recovery of joint function in these conditions (33–35). In 2015, Guoying et al. (31) found that the combination of TCM fumigation and functional exercise for post-stroke hemiplegic shoulder pain was more effective than functional exercise alone in relieving shoulder joint pain, improving joint mobility, and enhancing the quality of life. The results of the above studies are consistent with the findings of the present study, which shows that the addition of TCM fumigation treatment as an adjunct to conventional conservative treatment methods can effectively restore shoulder joint function in patients with rotator cuff injuries.

At the 2-month post-treatment follow-up, the experimental group showed statistically significant between-group improvements relative to the control group in the CMS score (MD = 6.8 points), VAS score (MD = −0.7 points), and modified UCLA score (MD = 2.1 points). However, all observed mean differences were below their respective minimal clinically important difference (MCID) thresholds-−10.0 points for CMS, 1.0 point for VAS, and 3.0 points for the modified UCLA score. While statistically significant differences were observed across the scoring groups, a clear distinction must be drawn between statistical significance and clinical significance. Despite the attainment of a *p*-value <0.017, the effect size failed to meet the established MCID, indicates that the clinical significance of the therapeutic effect of TCM fumigation at this time point is limited. In contrast, no statistically significant between-group differences were detected at the 6-month follow-up, suggesting that the treatment effect attributable to TCM fumigation was not sustained beyond 6 months. Given the transient nature of these effects, we hypothesize that the maximal between-group divergence may have occurred immediately after treatment cessation—potentially exceeding MCID thresholds—though this remains speculative in the absence of assessments conducted at the immediate post-intervention time point.

The repair of supraspinatus tissue induced by hypertonic dextrose injection involves a long-term biological process, with structural reconstruction and functional recovery typically requiring several months. In the early stages of treatment, patients often fail to notice subjective symptom improvement; some individuals may even experience transient worsening of pain due to the stimulation of tendon tissue by the hypertonic environment, potentially affecting treatment adherence and confidence. Applying TCM fumigation therapy under these circumstances can rapidly alleviate shoulder joint pain and improve mobility within a short period, achieving patients satisfaction early in treatment and thereby enhancing treatment acceptance and willingness to adhere to long-term rehabilitation programs.

## 5 Study limitations

This study has a few limitations. First, this was single-center study conducted on a limited sample population of patients with rotator cuff injuries and based on a TCM formula that had been developed on the basis of long-standing clinical experience. Consequently, while the findings may be most generalizable to the Chinese population, their external validity, specifically for populations in other countries, is yet to be examined. Second, MRI serves as an objective modality for the assessment of rotator cuff injuries. However, we found that the improvement in shoulder joint function among participants was not positively correlated with radiological changes—a finding corroborated by the work of other researchers (36). Accordingly, we employed subjective observational measures, including the VAS score, CMS score, and modified UCLA score, to evaluate the degree of shoulder function improvement. This approach may potentially introduce bias into the expected outcomes. Third, the follow-up duration was relatively short, and long-term efficacy beyond 6 months could not be assessed. Fourth, double-blinding of both the subjects and the operators was not conducted in this study, which might lead to certain subjective expectation biases and affect the efficacy evaluation.

## 6 Conclusion

The effects of both treatment methods on subjects with rotator cuff injuries were significant. However, the study revealed that in the early treatment phase for patients with rotator cuff injuries, the combined approach of ultrasound-guided hypertonic dextrose injection followed by TCM fumigation showed greater efficacy than injection alone in improving shoulder joint range of motion, reducing pain scores, and restoring shoulder function, while maintaining a favorable safety profile. The improved treatment outcomes were sustained for at least 2 months. Nevertheless, there was no difference between the long-term efficacy of the two approaches at the end of 6 months after the treatment. Effective treatment of rotator cuff injuries can be planned according to the patient's individual preferences and suitability.

## Data Availability

The datasets presented in this study can be found in online repositories. The names of the repository/repositories and accession number(s) can be found below: The datasets analyzed during the current study are available from the Clinical Trial Public Management Platform (http://www.medresman.org.cn), under registration number ChiCTR2100050251, accessible directly at http://www.medresman.org.cn/pub/en/proj/search.aspx.
